# Benzyl butyl phthalate decreases myogenic differentiation of endometrial mesenchymal stem/stromal cells through miR-137-mediated regulation of PITX2

**DOI:** 10.1038/s41598-017-00286-6

**Published:** 2017-03-15

**Authors:** Hung-Sheng Chen, Chia-Yi Hsu, Yu-Chia Chang, Hui-Yu Chuang, Cheng-Yu Long, Tsung-Hua Hsieh, Eing-Mei Tsai

**Affiliations:** 1 Graduate Institute of Medicine, College of Medicine, Kaohsiung Medical University, Kaohsiung City, Taiwan; 2 Department of Obstetrics and Gynecology, Kaohsiung Medical University Hospital, Kaohsiung Medical University, Kaohsiung City, Taiwan; 3 Research Center for Environmental Medicine, Kaohsiung Medical University, Kaohsiung, Taiwan; 4 Center for Stem Cell Research, Kaohsiung Medical University, Kaohsiung, Taiwan; 5 Center for Infectious Disease and Cancer Research, Kaohsiung Medical University, Kaohsiung, Taiwan

## Abstract

Phthalate, an environmental toxin, has been considered as an endocrine-disrupting chemical. Growing evidence has demonstrated links between endocrine-disrupting chemicals, tissue development, and reproductive physiology, but the mechanisms of gene expression regulation by environmental factors that affect cell differentiation are unclear. Herein, we investigated the effects of butyl benzyl phthalate (BBP) on human endometrial mesenchymal stem/stromal cell (EN-MSC) differentiation and identified a novel signaling pathway. Differentiation of endometrial mesenchymal stem/stromal cells decreased after administration of BBP. We analyzed BBP regulation of gene expression in EN-MSC using cDNA microarrays and Ingenuity Pathway Analysis software to identify affected target genes and their biological functions. *PITX2* emerged as a common gene hit from separate screens targeting skeletal and muscular disorders, cell morphology, and tissue development. BBP decreased transcription of *PITX2* and elevated expression of the microRNA miR-137, the predicted upstream negative regulator of *PITX2*. These data indicated that BBP affects *PITX2* expression through miR-137 targeting of the 3′ untranslated region of *PITX2* mRNA. *PITX2* down-regulation also decreased *MyoD* transcript levels in EN-MSC. Our results demonstrate that BBP decreases EN-MSC myogenic differentiation through up-regulation of miR-137, contribute to our understanding of EN-MSC differentiation, and underline the hazardous potential of environmental hormones.

## Introduction

Human mesenchymal stem/stromal cells (hMSCs) are adult stem cells that maintain tissue homeostasis by serving as a source of renewable progenitor cells to repair injured tissues and replace cells in routine cellular turnover throughout adult life^1, 2^; they may be isolated from a variety of tissues. Human mesenchymal stem cells (MSCs) have been isolated from a variety of tissues, including bone marrow, blood, adiopose, endometrium and other adult tissues. Among the diverse origins, we used MSCs derived form endometrium tissues. The human endometrium is a highly regenerative tissue that undergoes menstrual cycles involving growth, differentiation, and shedding during a woman’s reproductive life. The differentiation ability of the endometrium is based on endometrial stem cells^3–5^. Therefore endometrial adult stem cell populations are thought to be responsible for this remarkable regenerative capacity^3, 4^. Endometrial mesenchymal stem/stromal cells (EN-MSCs) are multi-potent stem cells that may be isolated and induced *in vitro* to differentiate into a variety of cell lineages that include adipocytes, osteocytes, chondrocytes, and myocytes^5^. EN-MSC differentiation is controlled by regulatory genes that induce progenitor cell differentiation into a specific lineage; in addition, environmental factors, such as phthalates, may influence gene expression during cell differentiation^6^. However, how environmental factors affect cell differentiation through gene expression regulation is unclear.

The pollutant butyl benzyl phthalate (BBP) is ubiquitously present in the environment. BBP is widely used as a plasticizer in the polyvinyl chloride industry and is commonly found in a variety of products such as automotive trim, food packaging, medical products and children’s toys^7^. BBP is an external plasticizer, i.e., used in resin softening without chemical binding to the final product. Therefore, BBP tends to migrate slowly out of discarded plastics and disperse into aqueous environments^8, 9^; hence, BBP may enter the food chain^10^. In addition, phthalates have been classified as endocrine-disrupting chemicals (EDCs) and may interfere with the endocrine system to produce adverse developmental, reproductive, neurological, and immunological effects^11–13^. In previously study, Upson K *et al.* finding that urinary concentration of the BBP metabolite MBzP (mono-n-benzyl phthalate) may be associated with increased risk of endometriosis^14^. Reddy *et al*. has demonstrated the relationship between exposure to polyethylenes such as BBP and the occurrence of endometriosis in infertile women^15^.

MicroRNAs (miRNAs) are small, endogenous non-coding RNAs that regulate gene expression by forming imperfect base pairing to sequences in the 3′ untranslated regions (UTRs) of their target mRNAs, thereby triggering translational repression. miRNAs influence a variety of biological processes including development, tissue morphogenesis, cell growth, and maintenance of tissue identity^16^. Emerging evidence indicates that miRNAs have a critical role in the self-renewal and differentiation of MSCs^17^.

In addition to the contribution of genetic background, increasing evidence indicates that EDCs may affect MSC differentiation^18, 19^. Therefore, the aim of the present study was to evaluate the effect of BBP on the differentiation of EN-MSCs and to investigate the relationship between BBP and epigenetic-modulated gene expression.

## Results

### EN-MSCs differentiate into adipogenic, osteogenic, chondrogenic, and myogenic lineages

To investigate the differentiation potential of EN-MSCs, we cultured cells from endometrium under conditions that favored adipogenic, osteogenic, chondrogenic, or myogenic differentiation. Cytochemical and immunofluorescence staining and quantitative real-time PCR (qPCR) were performed to determine the capacity for EN-MSCs to undergo various lineage differentiations after induction. Adipogenic, osteogenic, or chondrogenic differentiation of EN-MSCs was identified by Oil Red O, Alizarin Red S, or Alcian Blue staining of the respective markers (Supplementary Fig. S1A and B). Subsequent qPCR analysis revealed increased expression of the respective adipogenic-, osteogenic-, and chondrogenic-specific markers FABP4, Runx2, and collagen II (Supplementary Fig. S1C).

Myogenic differentiation, as assayed by immunoreactivity for MyoD (last two panels; Supplementary Fig. S1A) and qPCR analysis of transcript levels of myogenic markers, was also increased (last one panel; Supplementary Fig. S1B). These data suggested that the endometrium contains cells that have MSC properties associated with multiple lineage differentiation.

### BBP decreases EN-MSC adipogenic and myogenic differentiation

**Figure 1:**
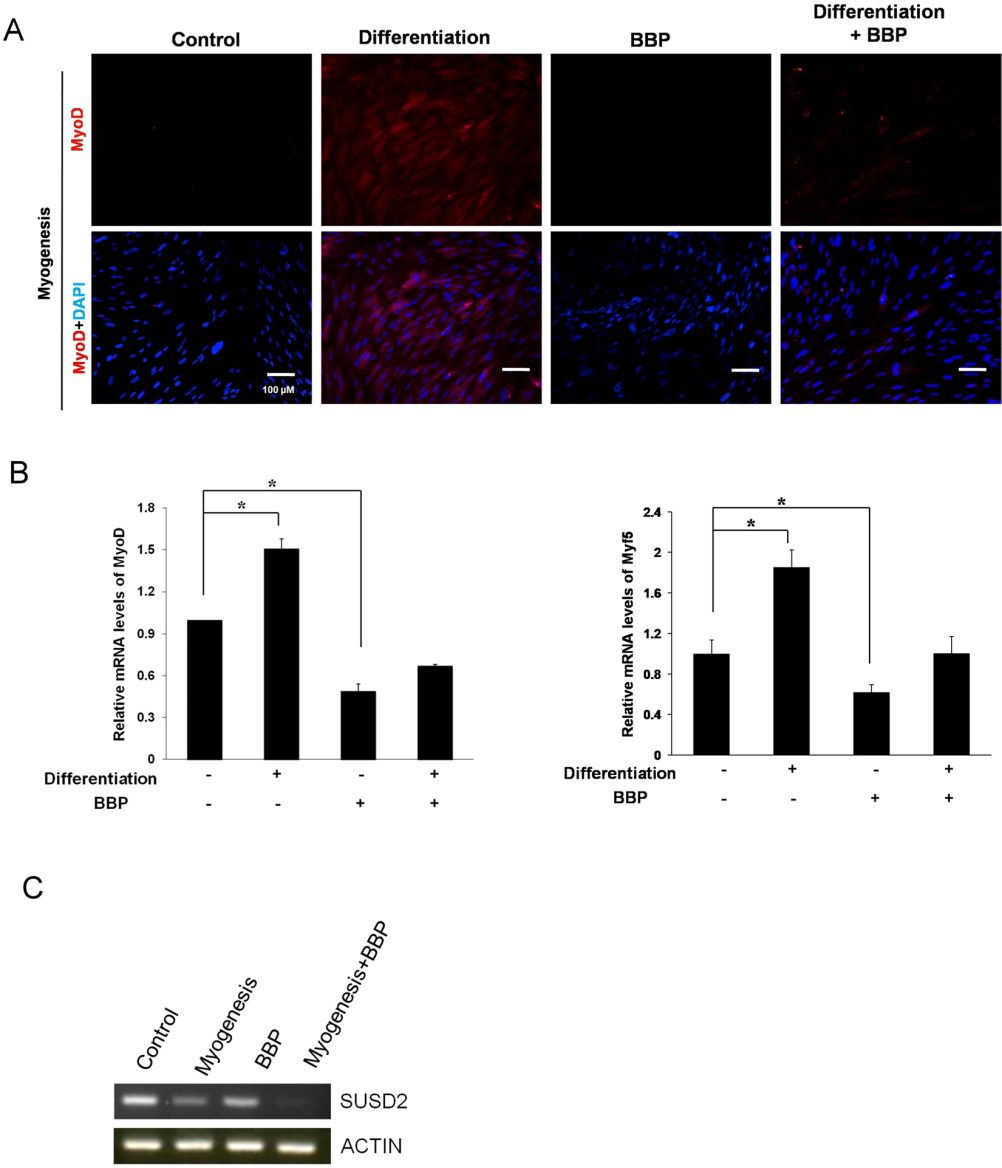
Effect of BBP on EN-MSC differentiation. (**A**) EN-MSCs were cultured in differentiation medium for 2 weeks and treated with or without 1 μM BBP every day. Staining and magnification were carried out as in (**A**). Differentiation is apparent in control differentiation samples, whereas there is signal reduction in the BBP-treated differentiation samples. (**B**) Gene expression analysis of myogenic markers in differentiated EN-MSCs by real-time PCR analysis. Expression was analyzed with qPCR, using 18S as an internal control. The BBP treatment protocol was as in (**A**). (**C**) The RNA extraction and PCR to detect the level of endometrial MSC markers *SUSD2*. The data shown represent the mean ± SD of three experiments with three different batches of cells. *P < 0.05.

To assess how BBP influences EN-MSC differentiation, cells were treated with 1 μM BBP during the induction period corresponding to multiple lineage differentiation. After EN-MSC differentiation induction and/or BBP treatment, adipogenic, osteogenic, chondrogenic, and myogenic differentiation was analyzed by cytochemical and immunofluorescence staining. In the absence of BBP treatment, EN-MSC differentiation after induction into the four lineages of interest was not affected; in contrast, in the presence of the BBP, EN-MSC differentiation was affected (Fig. 1A and Supplementary Fig. S2A and B). Further, we assessed whether BBP could influence the expression of marker genes during differentiation. BBP decreased the expression of the adipogenic marker *FABP4*, *PPARγ2* and myogenic marker *MyoD*, *Myf5* in each of the non-differentiated and differentiated condition (Fig. 1B and Supplementary Fig. S2C). These data revealed that BBP affected EN-MSC differentiation. We next examined the phenotype of BBP affected the myogenic differentiation of EN-MSC, we performed the RNA extraction and PCR to detect the level of endometrial MSC markers *sushi Domain Containing 2 (SUSD2)*. The data showed that the level of *SUSD2* markers was decreases in BBP treated EN-MSC, suggesting BBP affected EN-MSC differentiation through loss of the EN-MSC phenotype (Fig. 1C).

### cDNA microarray and signaling pathways

Next, we investigated how phthalate affected EN-MSC differentiation using whole-genome cDNA microarrays to examine BBP regulation of gene expression. For these experiments, we added 1 μM BBP to a culture of EN-MSCs for 24 h prior to the isolation of total RNA and subsequent cDNA synthesis. We focused the down-regulated gene after BBP-treatment in MSCs.

Table 1 lists the top 15 genes that were down-regulated after BBP treatment, which underscores the remarkable potential of this compound to alter EN-MSC differentiation. Analysis of cDNA microarray data revealed how individual genes interacted and coordinated to affect regulation of biological functions and signaling pathways. The top biofunctions associated with the gene expression profiles of BBP-treated MSCs were identified using Ingenuity Pathway Analysis and are listed in Supplementary Table S1: diseases and disorders, molecular and cellular functions, and physiological system development and function.

**Table 1:** Top 15 BBP-downregulated genes.

No.	Symbol	Fold change	Gene description
1.	*SLC5A12*	−130.750	solute carrier family 5, member 12
2.	*TDGF1*	−44.673	teratocarcinoma-derived growth factor 1
3.	*ALX1*	−39.616	ALX homeobox 1
4.	*VCY/VCY1B*	−35.254	Variable Charge, Y-Linked/Variable Charge, Y-Linked1B
5.	*LGI3*	−32.750	leucine-rich repeat LGI family, member 3
6.	*COL9A3*	−32.254	collagen, type IX, alpha 3
7.	*ANXA13*	−31.313	annexin A13
8.	*ADH4*	−20.607	alcohol dehydrogenase 4 (class II), pi polypeptide
9.	*BRWD1*	−18.950	bromodomain and WD repeat domain containing 1
10.	*MS4A1*	−18.920	membrane-spanning 4-domains, subfamily A, member 1
11.	*SSX3*	−4.42	Synovial Sarcoma, X Breakpoint 3
12.	*VSX1*	−0.109	visual system homeobox 1
13.	*ZIC3*	−0.258	Zic family member 3
14.	*IL13*	−0.387	interleukin 13
15.	*PTPRC*	−0.390	protein tyrosine phosphatase, receptor type, C

Finally, we identified the major biofunction categories associated with cellular differentiation. Table 2 lists the genes associated with skeletal and muscular disorders, cell morphology, and tissue development. Biofunctions of the candidate genes responsible for BBP-induced alterations of EN-MSC differentiation were determined.

**Table 2:** Functional description of candidate genes responsible for BBP regulation of EN-MSCs.

Skeletal and Muscular Disorders		Cell Morphology		Tissue Development	
Symbol	Gene description	Symbol	Gene description	Symbol	Gene description
*ALX1*	ALX homeobox 1	*ASIC2*	acid-sensing (proton-gated) ion channel 2	*ADAM28*	ADAM metallopeptidase domain 28
*CASP1*	caspase 1, apoptosis-related cysteine peptidase	*BCMO1*	beta-carotene 15,15′-monooxygenase 1	*ALX1*	ALX homeobox 1
*CASP192*	caspase 192	*CASP1*	caspase 1, apoptosis-related cysteine peptidase	*CDH1*	cadherin 1, type 1, E-cadherin (epithelial)
*CCL23*	chemokine (C-C motif) ligand 23	*CDH1*	cadherin 1, type 1, E-cadherin (epithelial)	*CDH24*	cadherin 24, type 2
*CDH1*	cadherin 1, type 1, E-cadherin (epithelial)	*ESRRG*	estrogen-related receptor gamma	*GLIS3*	GLIS family zinc finger 3
*CHEK2*	checkpoint kinase 2	*GHRL*	ghrelin/obestatin prepropeptide	*HIPK1*	homeodomain interacting protein kinase 1
*CHRM2*	cholinergic receptor, muscarinic 2	*GLIS3*	GLIS family zinc finger 3	*HYAL1*	hyaluronoglucosaminidase 1
*COL9A3*	collagen, type IX, alpha 3	*IGLL1*	immunoglobulin lambda-like polypeptide 1	*LHX9*	LIM homeobox 9
*FASLG*	Fas ligand (TNF superfamily, member 6)	*KIF18B*	kinesin family member 18	*OTX1*	orthodenticle homeobox 1
*GYPA*	glycophorin A (MNS blood group)	*MTMR3*	myotubularin related protein 3	*OVOL1*	ovo-like 1 (Drosophila)
*HIPK*	homeodomain interacting protein kinase 1	*NOBOX*	*NOBOX* oogenesis homeobox	*PITX2*	paired-like homeodomain 2
*HYAL1*	hyaluronoglucosaminidase 1	*PITX2*	paired-like homeodomain 2	*PTGER3*	prostaglandin E receptor 3 (subtype EP3)
*KCNE3*	potassium voltage-gated channel, Isk-related family, member 3	*PTGER3*	prostaglandin E receptor 3 (subtype EP3)	*PTPRC*	protein tyrosine phosphatase, receptor type, C
*KCNJ15*	potassium inwardly-rectifying channel, subfamily J, member 15	*PTPRC*	protein tyrosine phosphatase, receptor type, C	*RGS3*	regulator of G-protein signaling 3
*MDM2*	*Mdm2*, p53 E3 ubiquitin protein ligase homolog (mouse)	*RGS3*	regulator of G-protein signaling 3	*SRC*	v-*src* sarcoma (Schmidt-Ruppin A-2) viral oncogene homolog (avian)
*MS4A1*	membrane-spanning 4-domains, subfamily A, member 1	*SRC*	v-*src* sarcoma (Schmidt-Ruppin A-2) viral oncogene homolog (avian)	*TCF12*	transcription factor 12
*OTX1*	orthodenticle homeobox 1	*TLR2*	toll-like receptor 2	*TDGF1*	teratocarcinoma-derived growth factor 1
*PHACTR3*	phosphatase and actin regulator 3	*ZP3*	zona pellucida glycoprotein 3 (sperm receptor)	*TLR2*	toll-like receptor 2
*PITX2*	paired-like homeodomain 2			*WWOX*	WW domain containing oxidoreductase
*PTPRC*	protein tyrosine phosphatase, receptor type, C			*ZP3*	zona pellucida glycoprotein 3 (sperm receptor)
*SLC22 A12*	solute carrier family 22 (organic anion/urate transporter), member 12				
*SRC*	v-*src* sarcoma (Schmidt-Ruppin A-2) viral oncogene homolog (avian)				
*TLR2*	toll-like receptor 2				
*TPIM10*	?				
*WWOX*	WW domain containing oxidoreductase				
*ZNF*	zinc finger protein				

### Gene expression in BBP-treated MSCs

**Figure 2:**
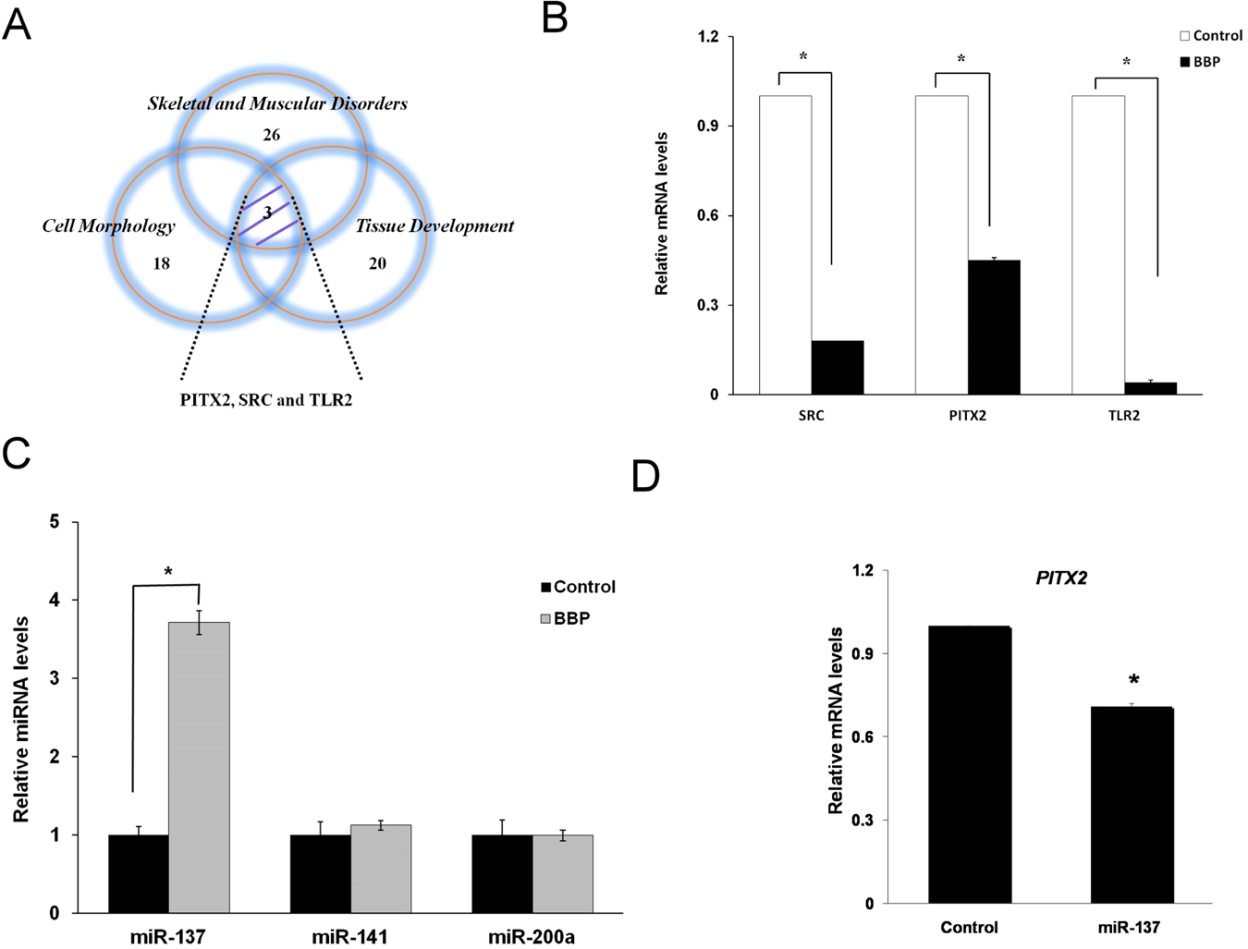
mRNA levels of the three identified genes. (**A**) Venn diagram with the number of genes differentially expressed between biofunctions in three individual categories: skeletal and muscular disorders, cell morphology, and tissue development. The shaded area shows intersection of all three categories and denotes three shared genes that emerged: *PITX2*, *SRC*, and *TLR2*. (**B**) qRT-PCR analysis of mRNA levels of the three identified genes; all genes showed significant reduction in expression after BBP treatment as compared with controls. (**C**) The level of miR-137 was increased in BBP-treated EN-MSCs compared with control cells. U6 was detected as an internal control. (**D**) Validation of miR-137 target. Precursor-miR-137 effectively decreased the transcript level of *PITX2*. The data shown represent the mean ± SD of three experiments with three different batches of cells. *P < 0.05.

The gene was found to have overlapping contributions among the BBP deregulated genes whose functions were associated with skeletal and muscular disorders, cell morphology, and tissue development and were excluded which are irrelevant to MSCs from intersection of all three categories. We found the three genes, namely, PITX2 (fold change = 0.596, P = 0.00007), SRC (fold change = 0.532, P = 0.000082), and TLR2 (fold change = 0.645, P = 0.00078) (Fig. 2A). First, we examined gene expression levels of these three genes in EN-MSCs after 1 μM BBP treatment for 24 h. The data revealed that expression of the three genes decreased in BBP-treated MSCs (Fig. 2B). These data suggested that *PITX2*, *SRC*, and *TLR2* play a vital role in mediating the effects of BBP on EN-MSC differentiation. In previous studies, PITX2, a homeodomain transcription factor, is essential for normal development and differentiated of tissue^20, 21^. Recent studies have reported that SRC plays a role in signal pathways involved in cell proliferation, growth, survival osteoclast and intestinal epithelial cell differentiation^22, 23^. In addition, TLR2 play a central role in the innate immune system and is associated with B cell differentiation^24^. We then investigated whether miRNAs that target *SRC*, *PITX2*, and/or *TLR2* mRNAs mediate the effects of BBP on EN-MSC differentiation. We targeted *PITX2*, which has been reported to be associated with myogenesis^25–28^. We attempted to identify miRNAs that serve as upstream regulators of EN-MSC differentiation, i.e., miRNAs that might be affected by BBP; specifically that were affected by BBP in EN-MSC differentiation, and specifically we used the prediction software miRanda (http://www.microrna.org/) to select three candidate miRNA regulators of *PITX2*: miR-137, miR-141 and miR-200a. BBP treatment increased the level of miR-137 in EN-MSCs, whereas the levels of miR-141 and miR-200a were not affected (Fig. 2C). Next, we examined whether *PITX2* expression was affected by miR-137. Overexpression of precursor-miR-137 in EN-MSCs reduced the *PITX2* transcript level (Fig. 2D). These data suggested that the BBP-induced effects on the level of *PITX2* transcript are mediated through miR-137.

### miR-137 down-regulates *PITX2* by targeting the 3′UTR

**Figure 3:**
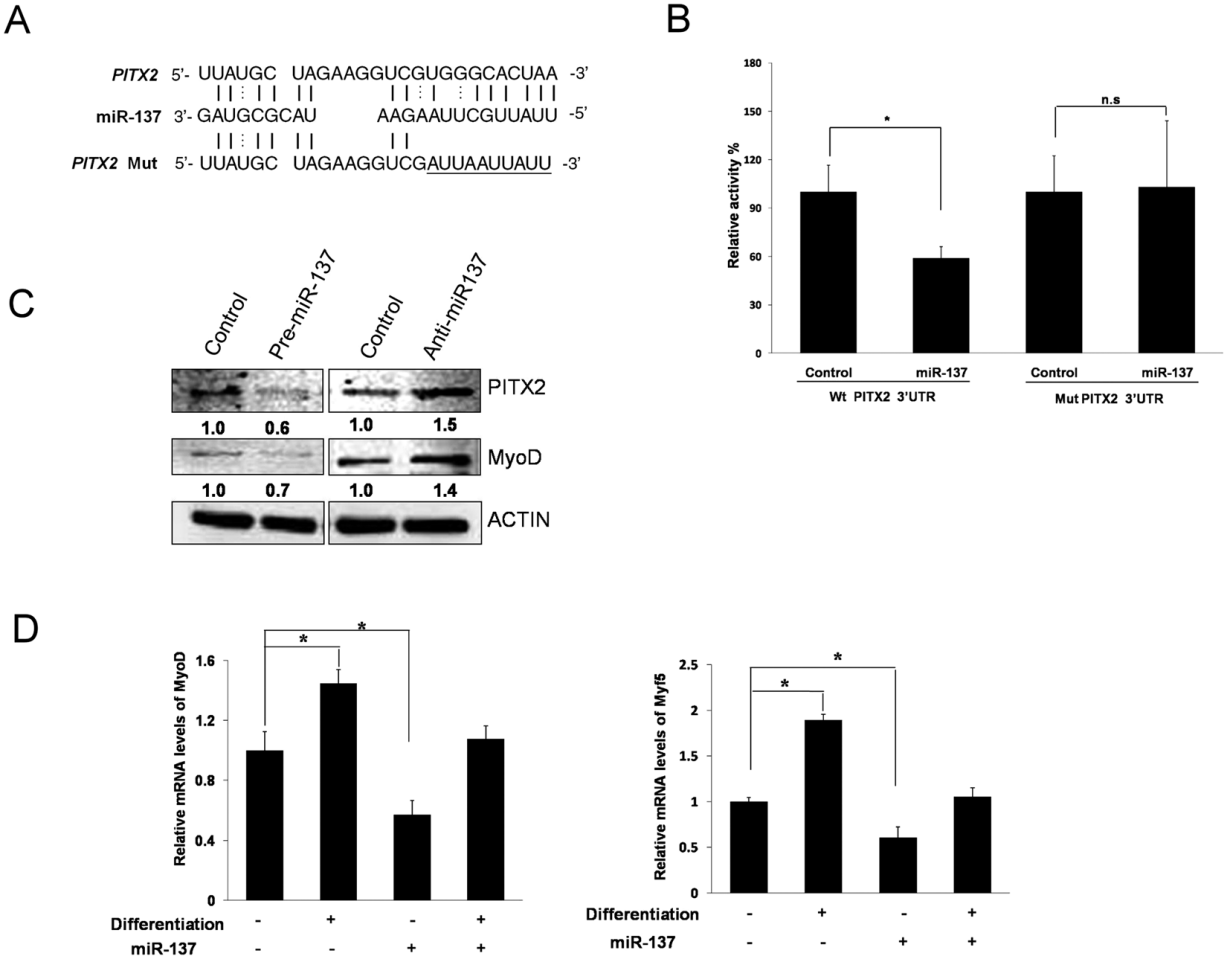
*PITX2* mRNA is a direct target of miR-137. (**A**) Sequences of target sites for miR-137 in the wild-type and mutant (Mut) versions of 3′-UTR of *PITX2* mRNA. (**B**) Cells were co-transfected with precursor-miR-137 or precursor control and the pGL2 vector containing wild-type (Wt) or mutant version of the putative *PITX2* 3′UTR miR-137 binding site. Luciferase activity was normalized to the control. (**C**) Western blot analysis was used to detect the expression of MyoD and PITX2 in response to miR-137 alteration. (**D**) EN-MSCs were cultured in differentiation medium for 2 weeks and transected with miR-137. Gene expression analysis of myogenic markers in differentiated EN-MSCs by real-time PCR analysis. Expression was analyzed with qPCR, using 18S as an internal control. The data shown represent the mean ± SD of three experiments with three different batches of cells. *P < 0.05.

To test whether miR-137 targets *PITX2*, the 3′UTR of *PITX2*, which contains a miR-137 binding site, was cloned into the pGL-2 control vector to create a luciferase reporter system (Fig. 3A). Co-transfection was performed with pre-miR-137 (precursor control) and pGL2-PITX2 3′UTR (mutant version of pGL2-PITX2 3′UTR). Cotransfection was performed with pre-miR-137 (precursor control) and either wild-type pGL2-PITX2 3′UTR or a mutant derivative. The luciferase reporter showed that miR-137 inhibited the *PITX2* wild-type reporter but did not affect the *PITX2* mutant reporter (Fig. 3B).

Further, we performed western blotting to confirm whether miR-137 affects the protein level of PITX2 and MyoD. Over-expression of miR-137 decreased the level of PITX2 and MyoD, whereas knock-down of mir-137 increase the levels of PITX2 and MyoD (Fig. 3C). These results indicated that, in our experimental system, *PITX2* was indeed a direct target of miR-137.

Taken together, these results showed that BBP reduced *PITX2* expression in EN-MSC differentiation (Fig. 2B) via increased expression of miR-137 (Fig. 2C), its upstream negative regulator. Therefore, we investigated whether myogenesis was affected by miR-137 in EN-MSCs. Ectopic miR-137 expression decreased the expression of the myogenic marker MyoD, PITX2 in differentiated condition (Fig. 3D), which supports the hypothesis that BBP exerts its effect on EN-MSC myogenic differentiation through the action of miR-137.

### miR-137 affects myogenesis through *PITX2*

**Figure 4:**
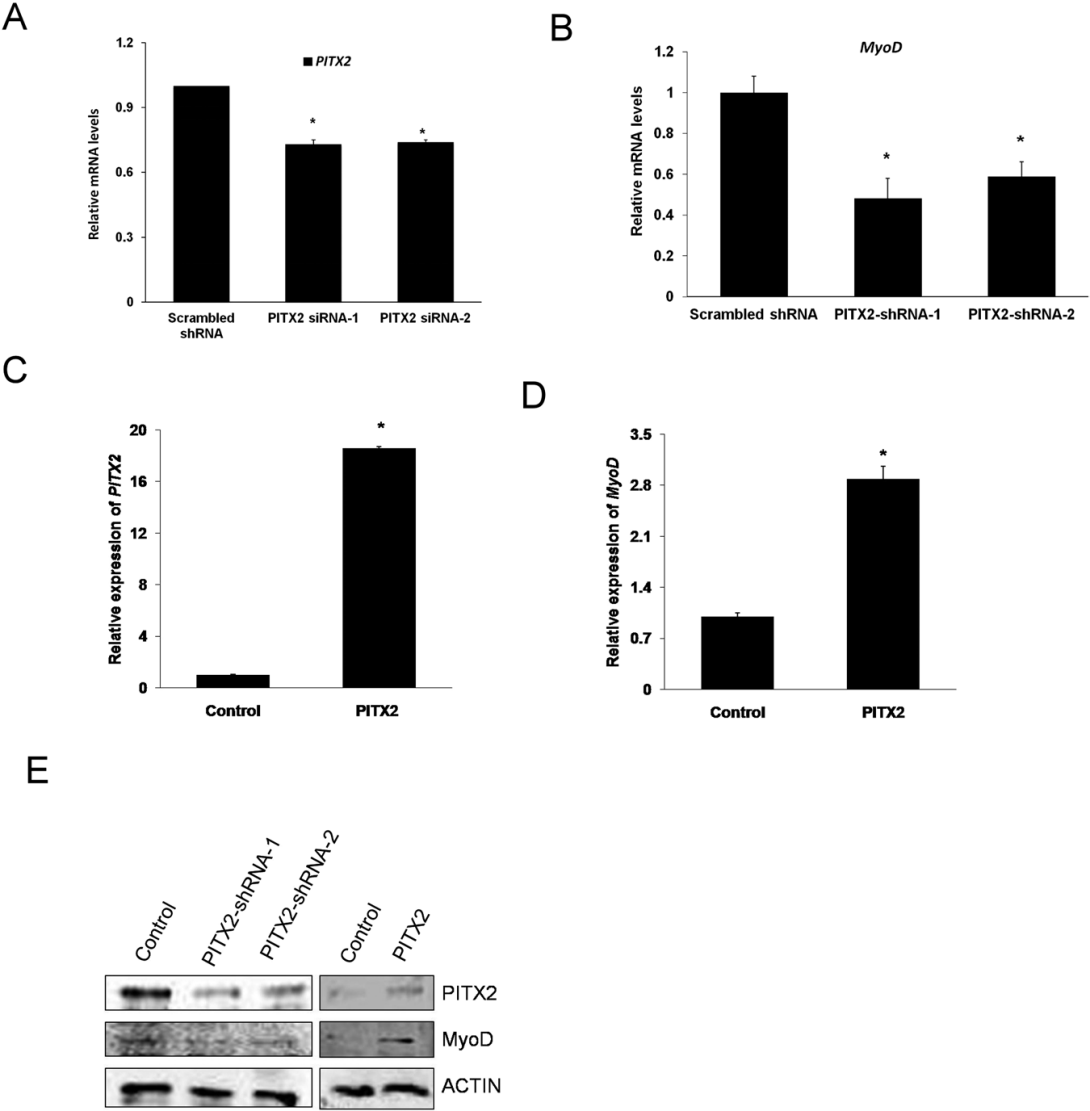
Knockdown and overexpression of *PITX2* affect *MyoD* expression. (**A**) EN-MSCs transfected with *PITX2* shRNA-1, *PITX*2 shRNA-2 or scrambled shRNA (a negative control for *PITX2* shRNA). (**B**) qPCR analysis of mRNA levels of *MyoD*. Expression levels were normalized to 18S rRNA levels. (**C**) EN-MSCs were transfected with *PITX2* or control vector. qPCR analysis of mRNA levels of *PITX2*. (**D**) mRNA levels of *MyoD* were analyzed with qPCR. Expression levels were normalized to 18S rRNA levels. (**E**) Western blotting indicated that PITX2 and MyoD level were positively correlated. The data shown represent the mean ± SD of three experiments with three different batches of cells. *P < 0.05.

To understand the extent to which miR-137 affects EN-MSC differentiation through *PITX2* directly, we used a short hairpin RNA (shRNA) to knockdown *PITX2* expression (Fig. 4A). When EN-MSC was transfected with PITX2-shRNA-1 or PITX2-shRNA-2, PITX2 and MyoD expression were down-regulated (Fig. 4B and E). PITX2 overexpression (Fig. 4C) in the cells increased PITX2 and MyoD expression (Fig. 4D and E). These data confirmed that PITX2 expression level had a significant effect on EN-MSC myogenic differentiation.

## Discussion

Phthalates are omnipresent toxins in the environment, and they have been classified as EDCs that can interfere with elimination of natural hormones that are responsible for homeostasis and essential for growth and development^29^. In previously study, Upson K *et al.* finding that urinary concentration of the BBP metabolite MBzP (mono-n-benzyl phthalate) may be associated with increased risk of endometriosis^14^. Reddy *et al*. has demonstrated the relationship between exposure to polyethylenes such as BBP and the occurrence of endometriosis in infertile women^15^. These speculate the correlation between the exposures of BBP and endometrial diseases. In addition, the effects of exposure to BBP and its main metabolite MnBP (mono-n-butyl phthalate) and MBzP may have same effect in animal model^30, 31^. The pattern of malformations produced by MnBP was similar to that produced by BBP^31^. Previous study also reported that BBP, MnBP and MBzP caused embryolethality and malformations in mice^30^.

MSCs play an important role in tissue homeostasis, serving as a source of renewable progenitor cells to replace or repair tissue cells throughout adult life^32, 33^. In the present study, we found that BBP decreased EN-MSC differentiation. We identified one such target gene *PITX2*, which is a homeobox transcriptional factor that regulates muscle development^21, 34^. Normally, *PITX2* and *MyoD* transcription levels increase during myogenic differentiation^21^; thus, the observed reduction in *PITX2* and *MyoD* transcripts offers further evidence that BBP alters transcriptional regulation during stem cell differentiation in endometrial tissues. In addition, PITX2 is essential for development of multiple organs, including the lung, heart and pituitary gland^35^. Therefore, environmental hormones might affect tissue development through PITX2.

In the present study, we used microarray analysis to identify genes whose expression levels were altered by BBP. Our results found that *TLR2* and *SRC* were dysregulated in response to BBP treatment. This finding is consistent with previous studies, we found TLR2 gene related to immune^24^, and SRC which has been described to be related with epithelial cell differentiation^23^. A previous study has investigated the alteration in the gene expression following phthalate treatment in which exposure to this compound caused a dys-regulation in the expression of many genes, including apoptosis-, cell proliferation-, and immune response- related genes^36, 37^.

TLR2 plays a key role in immune system and is found in immune cell, such macrophages, B cell and mast cells^24^. MSC display unique suppressive properties on T-cell immunity, since TLR expressed on human MSC enhanced the immunosuppressive phenotype of MSC^38, 39^. Immunosuppressive properties of MSC most probably depend on environmental factors^40^. Interestingly, functional role of phthalate-elicited differential gene expression is associated with immune system. It may be informative to investigate the potential mechanism related to BBP effects on immune system.

In the present study, we investigated how phthalate affected EN-MSC differentiation using whole-genome cDNA microarrays to examine BBP regulation of gene expression. Although the top 15 down-regulated genes were not found to have overlapping contributions among the BBP deregulated genes whose functions were associated with skeletal and muscular disorders, cell morphology, and tissue development. However, some studies have reported that these genes, TDGF1^41^, ALX1^42^, LGI3^43^, ADH4^44^, VSX1^45^, ZIC3^46, 47^, are relevant to stem cell differentiation or tissue development. As expected, phthalate exposure might affect cell differentiation or development.

Recently, several studies showed that exposure to various environmental or growth factors regulates the expression of certain specific miRNAs^48, 49^. Although several studies have demonstrated that miR-137 functions in neurogenesis or adipogenesis in stem cells^50, 51^, ours is the first study to demonstrate that BBP administration modulates miR-137 level and to identify PITX2 as a novel miR-137 downstream target during myogenic differentiation.

In conclusion, we characterized the roles of miR-137 in myogenic hMSC differentiation and elucidated the mechanisms of BBP action in this process. These findings contribute to our understanding of hMSC differentiation and underscore the hazardous potential of environmental hormones.

## Methods

### Cell line

EN-MSCs were isolated and collected from 3 different endometrium biopsies after hysterectomy for non-endometrial benign pathological condition, such as uterine prolapse. These women had not taken exogenous hormones for three months prior to surgery. This study was approved by the Institutional Review Board of Kaohsiung Medical University, and informed consent was obtained from each patient (KMUH-IRB-20140031). All experiments were performed in accordance with relevant guidelines and regulations. Written informed consent was obtained from each participant. EN-MSCs were isolated and purified as described^5^. Briefly, endometrial tissue was minced with sterile scissors and subjected to enzymatic digestion with 1 mg/ml type II collagenase for 60 to 90 minutes. After digestion, these digested tissues were filtered by wire sieves with serial different pores (100 μm, 70 μm and 40 μm diameter pores) to remove epithelial cells. These endometrial stromal cells were collected. For EN-MSC isolation, endometrial stromal cells (passage 5) were seeded in triplicate at clonal density; 200 cells per 100 mm Petri dish. After incubation of 21 days, large colonies were isolated and separated into single suspended cells by trypsinization. These cells were diluted and seeded in a 96-well plate, density at one cell per well. After incubation of 14 days, proliferated cells (which were from one single cell) were trypsinized and cultured in a 100 mm Petri dish. These early-passage EN-MSCs were used in the following experiments. EN-MSCs were characterized using MSC phenotypes and differentiation induction (i.e., adipogenesis, osteogenesis, and chondrogenesis,) and by gene expression, i.e., POU5F1 (previously known as OCT-4), CD29, CD44, CD49f, CD90, CD105, CD146, CD140b, and SUSD2 by flow cytometry^5^ (Supplementary Material). EN-MSCs were cultured in modified MCDB 153 medium (Keratinocyte-SFM, Gibco-Life Technologies, Carlsbad, CA) and Dulbecco’s Modified Eagle’s Medium: Nutrient Mixture F12 (Gibco-Life Technologies) (1:2, v/v) supplemented with 10% fetal bovine serum (Gibco-Life Technologies), 2 mM N-acetyl-l-cysteine (A8199, Sigma-Aldrich, St. Louis, MO) and 0.2 mM l-ascorbic acid 2-phosphate (Asc 2P; A8960, Sigma-Aldrich), and incubated at 37 °C in a humidified atmosphere with 5% CO_2_.

### Differentiation experiments

EN-MSCs were seeded at 5 × 10^4^ cells per well in a 6-well plate; differentiation conditions were applied the following day. Adipogenic differentiation of EN-MSCs was induced by treatment with 500 μM of 3-isobutyl-1-methylxanthine (I7018, Sigma-Aldrich), 1 μM dexamethasone (D8893, Sigma-Aldrich), 1 μM indomethacin (I8280, Sigma-Aldrich), and 10 μg/mL insulin (I1882, Sigma-Aldrich) (IDI-I medium) for 2 d, and followed by insulin treatment for 1 d. After 4 cycles of treatment over 12 d, the cells were fixed with 4% paraformaldehyde and stained with 0.2% Oil Red O for 30 min (O0625, Sigma-Aldrich)^52^. For osteogenic differentiation, 10 nM dexamethasone (D8893, Sigma-Aldrich), 50 μM Asc-2P, and 10 mM β-glycerophosphate disodium (G9891, Sigma-Aldrich), also commonly known as DAG medium, were added to the growth medium for 2 weeks. Medium changes and treatments were renewed once every 3 d^52^. At 14 d after the initiation of differentiation, the cells were stained with 2% Alizarin Red S (A5533, Sigma-Aldrich) to assay for osteocytes^53^. For myogenic differentiation, 5 μM 5-azacytidine (A2385, Sigma-Aldrich) was added to the growth medium for 24 h, after which the myogenic induction medium was replaced with normal growth medium^54^. The medium was changed every 3 d for the remainder of the culture. On day 14 after induction of myogenic differentiation, cells were fixed in 4% paraformaldehyde, stained for MyoD (ab64159, Abcam), and then examined for the presence of myocytes by immunofluorescence. After reaching 90% confluence, the cells were harvested and reseed in 15 ml tube at 2.5 × 10^5^ cells/tube. Chondrogenic differentiation of EN-MSCs was induced by treatment with 10 ng/mL TGF-β1 (T1654, Sigma-Aldrich), 50 μM Asc-2P, and 6.25 μg/mL insulin (TAI medium) in the 24 well plates. Medium was changed every 3 d^52, 55, 56^. After 14 d, the micromass was fixed in 4% paraformaldehyde and then examined for chondrocytes by staining with, 1% Alcian Blue 8-Gx, pH 1.0 (A5268, Sigma-Aldrich).

### Chemicals and Reagents

BBP (98%) was purchased from Sigma-Aldrich and diluted with ethanol to a concentration 1000-fold higher than the final concentration that was used in cell culture.

### quantitative real-time PCR analysis

qPCR was used to assess gene and miRNA expression. RNAs were extracted from EN-MSCs using TRI Reagent (Sigma-Aldrich). Reverse transcription was carried out with 1.5 μg of RNA using the Deoxy + HiSpec RT kit (Yeastern, Taipei, Taiwan) and TaqMan MicroRNA Reverse Transcription kit (Applied Biosystems, Foster City, CA). The expression of various transcripts and mature miRNAs was assessed by real-time PCR with Power SYBR Green PCR Master Mix (Applied Biosystems) and TaqMan MicroRNA Assay using an ABI 7900 Real-Time PCR system (Applied Biosystems). The primer sets used in this study are listed in Supplemental Table S4. Changes in gene expression were calculated relative to 18S RNA using the 2^−ΔΔCt^ method. MiRNAs expression were normalized to endogenous small nuclear U6B RNA using the 2^−ΔΔCt^ method.

### Immunofluorescence

After treatment, EN-MSCs were rinsed several times with PBS and fixed in 4% paraformaldehyde for 5 min, permeabilized with 0.5% Triton X-100 in PBS for 5 min. The fixed cells were probed with Rabbit-MyoD antibody (1:1000, ab64159, Abcam) and secondary antibody followed by Alexa Fluor 568–conjugated goat anti-rabbit IgG (1:500, A11011, Gibco-Life Technologies) for 45 min. Nuclei were counterstained with 4,6-diamidino-2-phenylindole (DAPI, 1 μg/ml, Roche). Images were obtained using a fluorescence microscope (Nikon Eclipse TE 300, Tokyo, Japan).

### cDNA microarray and data analysis

RNA was extracted from EN-MSCs using TRI Reagent. RNA integrity number >7.0 were used to synthesize the first strand cDNA via reverse transcription using an Illumina Total Pre RNA Amplification Kit (Ambion, Austin, TX, USA). Amplified cRNA samples were hybridized with streptavidin-Cy3 and scanned on the Illumina Beadstation GX. To determine differentially expressed genes, microarray data (n = 2 in each group) were analyzed using the gene expression module in Illumina Beadstudio software, version 3.3.7. Intensity data were normalized using the Beadstudio cubic spline algorithm and calculated with Beadstudio software according to the manufacturer’s protocols. The gene expression fold change of the stimulated cells was calculated as the average signal value relative to the average signal value for the control cells. Genes were selected based on a p-value cut-off (after adjustment) of p < 0.05 to control the false discovery rate (FDR)^57, 58^. A significant down-regulation was defined as a foldchange ≥1.5. We applied the Ingenuity Pathway Analysis (Ingenuity Systems, Redwood City, California) tool for analysis of canonical pathways and participating networks using the cDNA microarray data.

### Ingenuity Pathway Analysis

The molecular functions of the unique gene analysis of the BBP-induced genes were performed using Ingenuity Pathway Analysis (IPA) software (IPA, Ingenuity Systems, Redwood City, California). Genes from the data set that met the cutoff of and were associated with biological functions and/or diseases in the Ingenuity Pathways Knowledge Base were included in the analysis.

### Transfection

Transfection of each of miRNAs, shRNA, plasmid DNA, and reporter vectors was performed using TransIT-LT1 Transfection Reagent (Mirus Bio, Madison, WI). The following plasmids were used: Precursors of miR-137 and anti-miR-137 plasmids were purchased from System Biosciences. *PITX2* plasmid DNA was from the Bioresource Collection and Research Center, Hsinchu, Taiwan. The shRNAs included shRNA-PITX2#1 (TRCN0000020481), shRNA-PITX2#2 (TRCN0000235583), and scrambled control shRNA (TRCN0000040032) (National RNAi Core Facility at the Institute of Molecular Biology, Academia Sinica, Taipei, Taiwan). Cells were harvested 2 d after transfection.

### Luciferase assay

Cells were seeded onto 48-well culture plates and co-transfected with 200 ng of vector pGL2-PITX2–3′UTR or pGL2 that contained a mutant version of the *PITX2* 3′UTR, 200 ng pre-miR-137 or a precursor control, 30 ng luciferase reporter, and 5 ng Renilla luciferase reporter. Luciferase activity was measured by the Dual-Luciferase Reporter Assay system (Promega, Madison, WI). Firefly luciferase activity was normalized to Renilla luciferase activity for each sample. The luciferase signal was read with a TD-20/20 luminometer (Turner Biosystems, Sunnyvale, CA).

### Western Blot

The proteins were extracted with RIPA lysis buffer (Millipore, Billerica, MA, USA) containing several protease and phosphatase inhibitors (GBiosciences, St Louis, MO, USA). The protein content was determined by a Bio-Rad Protein Assay system (Bio-Rad, Hercules, CA, USA). Equal amounts of protein were separated by 10% SDS-PAGE and transferred to PVDF membranes (Millipore, Bedford, MA, USA). Then the membrane was incubated with primary antibodies: anti-MyoD (ab126726, 1:1000, abcam), anti-PITX2 (ab55599, 1:1000, abcam), and anti-actin (1:5000; Sigma-Aldrich). The secondary antibodies used were goat-anti-mouse or anti-rabbit IgG conjugated to HRP (Santa Cruz Biotechnology), and the ECL reagents (Millipore) were used for immunodetection

### Statistical Analysis

Statistical analyses were performed using One-way ANOVA followed by Tukey’s HSD test for comparing differences between multiple groups and Student’s t-test for comparing differences between two groups. Data represented the mean ± standard deviation. *P* values < 0.05 were considered statistically significant.

## Electronic supplementary material

Supplementary Information
