# Supplementary material for: Benzyl butyl phthalate decreases myogenic differentiation of endometrial mesenchymal stem/stromal cells through miR-137-mediated regulation of PITX2
Source: Sci Rep. 2017 Mar 15;7:186. doi: 10.1038/s41598-017-00286-6 (PMC5428022; doi:10.1038/s41598-017-00286-6)
Supplement: Supplementary file 1 — Supplementary Information [file 41598_2017_286_MOESM1_ESM.doc]

*Scientific Reports*

**Supplementary information**

**Benzyl butyl phthalate decreases myogenic differentiation of endometrial mesenchymal stem/stromal cells through Mir-137-mediated regulation of PITX2**

**Hung-Sheng Chen^1,2*^, Chia-Yi Hsu^2*^, Yu-Chia Chang^1^, Hui-Yu Chuang^2^, Cheng-Yu Long^2^, Tsung-Hua Hsieh^2#^,Eing-Mei Tsai^1,2,3,4,5#^**

^1^Graduate Institute of Medicine, College of Medicine, Kaohsiung Medical University, Kaohsiung City, Taiwan.

^2^Department of Obstetrics and Gynecology, Kaohsiung Medical University Hospital, Kaohsiung Medical University, Kaohsiung City, Taiwan.

^3^Research Center for Environmental Medicine, Kaohsiung Medical University, Kaohsiung, Taiwan.

^4^Center for Stem Cell Research, Kaohsiung Medical University, Kaohsiung, Taiwan. ^5^Center for Infectious Disease and Cancer Research, Kaohsiung Medical University, Kaohsiung, Taiwan

* H-S.C and C-Y.H contributed equally to this work.

^#^Correspondence: Department of Obstetrics and Gynecology, Kaohsiung Medical University Hospital, Kaohsiung Medical University, No. 100, Zihyou 1st Rd., Sanmin District, Kaohsiung City 807, Taiwan

Eing-Mei Tsai, E-mail: tsaieing@yahoo.com; Tsung-Hua Hsieh, E-mail: pelagice@yahoo.com.tw

**Supplementary material**

Flow Cytometric Analysis

EN-MSCs were stained with the following fluorochrome-conjugated antibodies, or their corresponding isotype controls, OCT4-PE (40/OCT3) purchased from BD Biosciences (San Jose, CA), and CD44-PE (BJ18), CD29-PE (TS2/16), CD140b-PE (18A2), SUSD5-PE (W5C5), CD34-PE (581), CD146-FITC (PTH12), CD90-FITC (5E10), CD105-FITC (43A3), CD45-FITC (2D1), SSEA4-FITC (MC-813-70) purchased from Biolegend, and CD49f-FITC (450-30A) purchased from Abcam. All antibodies were used at optimal concentrations. Cells were analyzed with a BD FACSCalibur cytometer and data were processed with CellQuest software (Becton Dickinson, San Jose, CA).


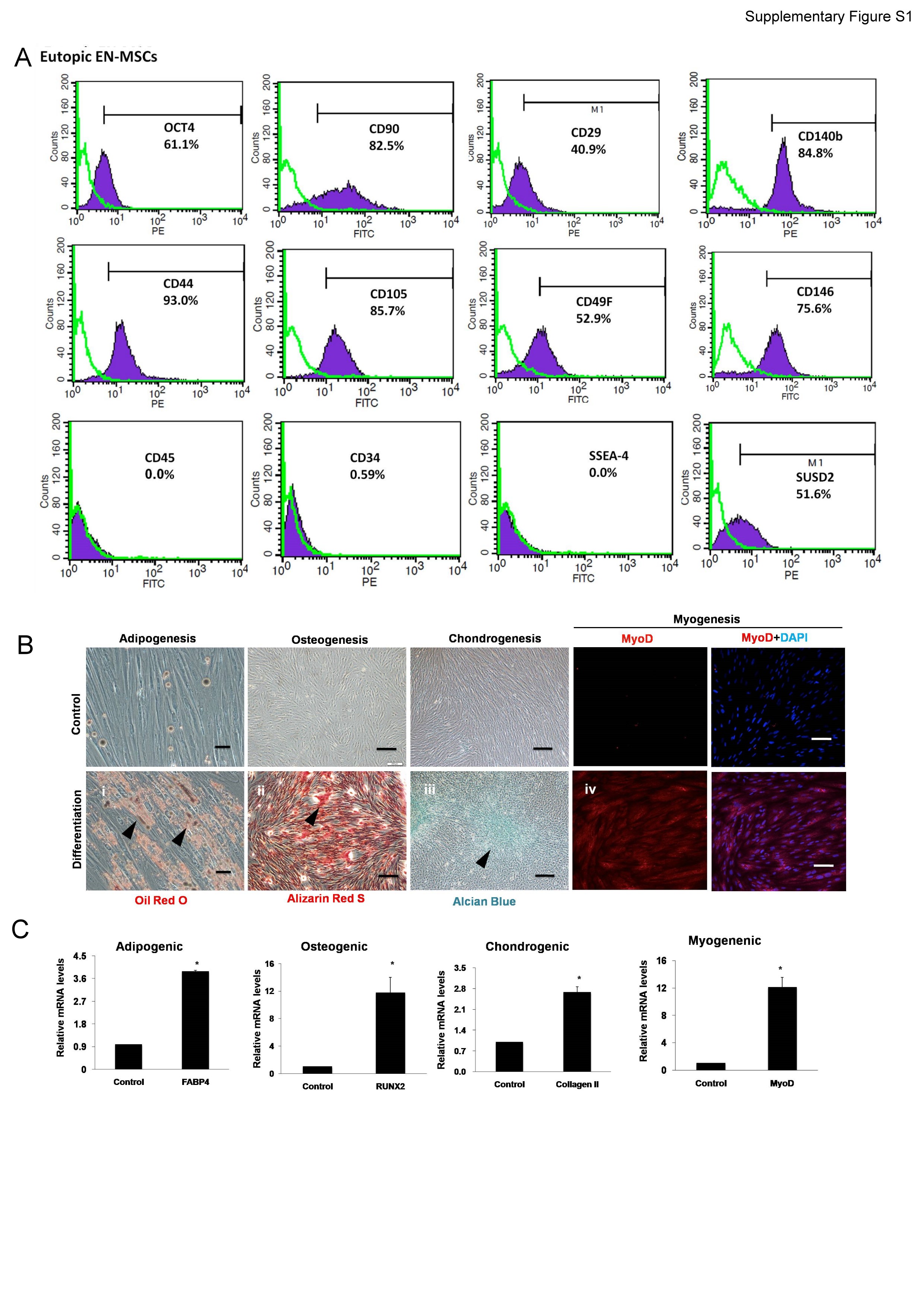


**Supplementary Figure S1. Induction of multilineage MSC differentiation**. (A) Characterization of eutopic human endometrial mesenchymal stem cells (EN-MSCs). Flow cytometry of EN-MSCs with mesenchymal stem cell, leukocytes cell, embryonic stem cell and hematopoietic stem cell markers. Mesenchymal stem cell markers (CD29, CD44, CD49F, CD90, and CD105), endometrial stem cell markers (CD146, CD140b and SUSD2) and stem cell marker (OCT4) were expressed whereas hematopoietic stem cell marker (CD34), leukocytes cell marker (CD45) and embryonic stem cell marker (SSEA4) were not. EN-MSCs were cultured in differentiation medium for 2 weeks. Cytochemical staining, immunofluorescence, and qPCR were used to determine EN-MSC differentiation. (B) i. Adipogenic differentiation shown by Oil Red O staining of lipid droplets (arrowheads; scale bar: 50 uM). ii. Osteogenic differentiation shown by Alizarin Red S staining of calcium (arrowhead; scale bar: 200 uM). iii. Chondrogenic differentiation shown by Alcian Blue staining of the extracellular matrix (arrowhead; scale bar: 200 uM). iv. Myogenic differentiation detected by immunostaining for MyoD; nuclei were stained with DAPI (scale bar: 100 uM). (C) Transcript level of the adipocyte gene *FABP4*, osteocyte gene *Runx2*, chondrocyte gene *Collagen II*, and myocyte gene *MyoD1* were measured by qPCR. 18S was used as loading control. Data show results from three independent experiments using different batches of cells (mean ± SD; n = 3). **P* < 0.05.


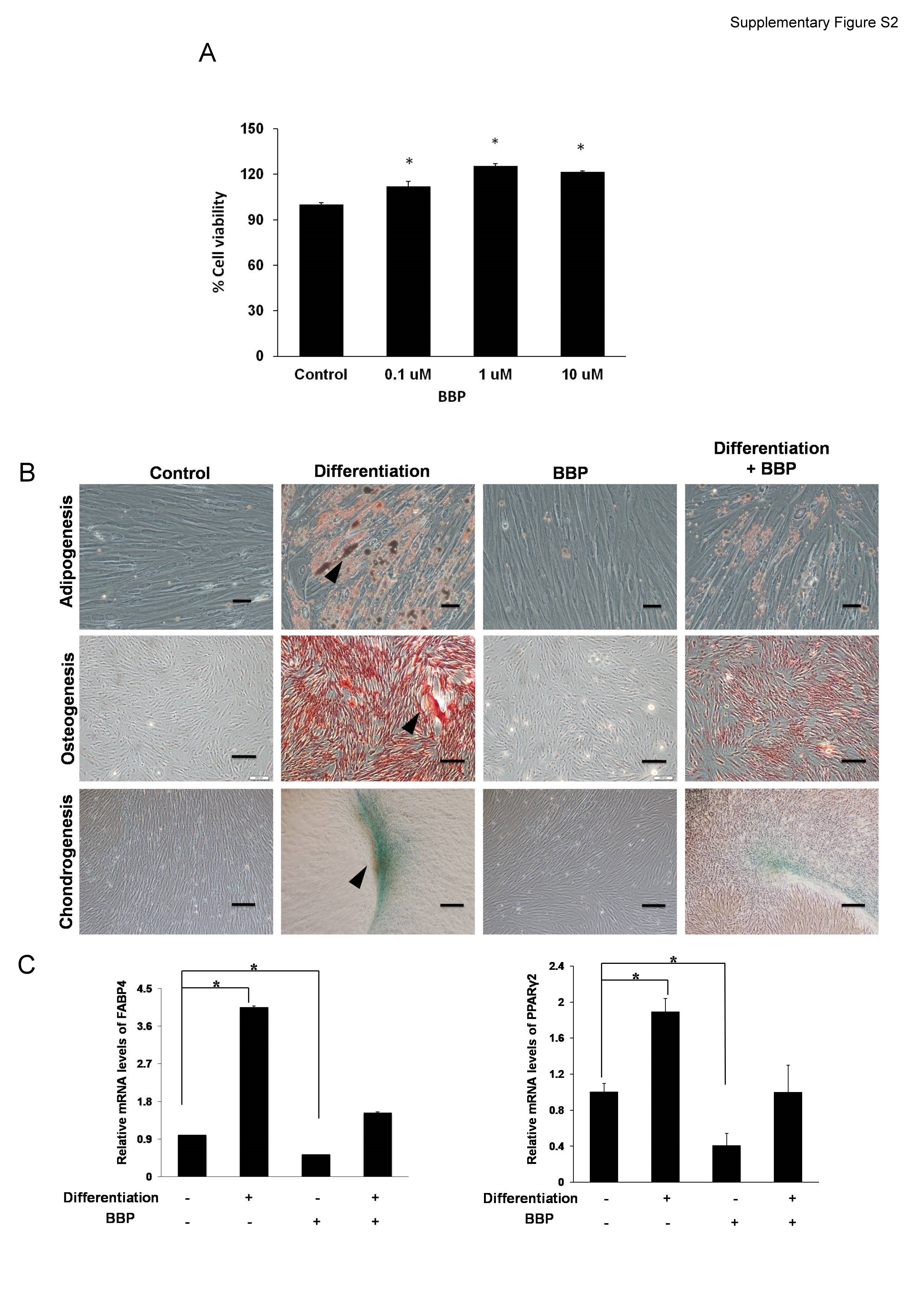


**Supplementary Figure S2. Effect of BBP on EN-MSC differentiation**. (A) WST-8 assay of cell proliferation in studies of dose dependence (0.1, 1, and 10 μM) in EN-MSCs treated with BBP. (B) EN-MSCs were cultured in differentiation medium for 2 weeks and treated with or without 1 μM BBP every day. Staining and magnification were carried out as in Supplementary Fig. S1A. Differentiation is apparent in control differentiation samples, i–iv, whereas there is signal reduction in the BBP-treated differentiation samples. (C) Gene expression analysis of adipogenic markers in differentiated EN-MSCs by real-time PCR analysis. Expression was analyzed with qPCR, using 18S as an internal control. The BBP treatment protocol was as in (A). Data show results from three independent experiments using different batches of cells (mean ± SD; n = 3). **P* < 0.05.

| Supplemental table 1. Top biofunctions. | | | |
| --- | --- | --- | --- |
| **Biofunctions** | ***P*-value** | **Number of Molecules** | |
| **Diseases and Disorders** |  | |  |
| *Gastrointestinal Disease* | 0.000471–0.0266 | | 10 |
| *Infectious Disease* | 0.000471–0.0266 | | 6 |
| *Respiratory Disease* | 0.000781–0.0266 | | 11 |
| *Dermatological Diseases and Conditions* | 0.00116–0.0241 | | 6 |
| *Skeletal and Muscular Disorders* | 0.00895–0.0157 | | 26 |
| **Molecular and Cellular Functions** |  | |  |
| *Cell-To-Cell Signaling and Interaction* | 0.0000566–0.0266 | | 21 |
| *Cell Death and Survival* | 0.00011–0.0266 | | 23 |
| *Cell Morphology* | 0.000471–0.0266 | | 18 |
| *Cell Cycle* | 0.00157–0.0266 | | 12 |
| *Cellular Growth and Proliferation* | 0.00162–0.0266 | | 25 |
| **Physiological System Development and Function** |  | |  |
| *Tissue Development* | 0.0000566–0.0266 | | 20 |
| *Embryonic Development* | 0.00139–0.0266 | | 22 |
| *Organ Development* | 0.00139–0.0266 | | 13 |
| *Organ Morphology* | 0.00139–0.0266 | | 27 |
| *Organismal Development* | 0.0013–0.0266 | | 14 |

| Supplemental Table 2. List of primer and shRNA sequences. | |
| --- | --- |
| Gene Name | Sequence |
| 18S | F:5'-GTAACCCGTTGAACCCCAT-3' |
|  | R:5'-CCATCCAATCGGTAGTAGCG-3' |
| FABP4 | F:5'-CTGGGCCAGGAATTTGACGAA-3' |
|  | R:5'-CATGACGCATTCCACCACCAG-3' |
| Runx2 | F:5'-GCACCGACAGCCCCAACTT-3' |
|  | R:5'-CCACGGGCAGGGTCTTGTT-3' |
| Collagen II | F:5'-GCACCCATGGACATTGGAGGG-3' |
|  | R:5'-GACACGGAGTAGCACCATCG-3' |
| MyoD | F:5'-GACAGGGAGGAGGGGTAGAG-3' |
|  | R:5'-TGCTGTCTCAAAGGAGCAGA-3' |
| SRC | F:5'-ACCTGGGAGGATGGGTTTT-3' |
|  | R:5'-GCACTCAAGAGGCCAGAACT-3' |
| PITX2 | F:5'-AGCCCGTCGCTGAATTCCGC-3' |
|  | R:5'-GGCCGGTCCACTGCATACTC-3' |
| TLR2 | F:5'-AAGGGAAGGGCTTAGCTTCA-3' |
|  | R:5'-TGGGTCGAAGAAGAGAGCTG-3' |
| U6 | F:5'- GTGCTCGCT TCGGCAGCACATATAC-3' |
|  | R:5'- AAA AAT ATG GAACGC TTC ACG AAT TTG-3' |
| SUSD2 | F:5'- AGAGCTGGATGGACCTGAAA-3' |
|  | R:5'- ATGCCAGCATGATGGAGAC-3' |
